# Growth Limiting pH, Water Activity, and Temperature for Neurotoxigenic Strains of *Clostridium butyricum*

**DOI:** 10.1155/2013/731430

**Published:** 2013-09-30

**Authors:** Hamid B. Ghoddusi, Richard E. Sherburn, Olusimbo O. Aboaba

**Affiliations:** ^1^Microbiology Research Unit, School of Human Sciences, London Metropolitan University, London N7 8DB, UK; ^2^Department of Food & Nutritional Sciences, The University of Reading, Whiteknights, Reading RG6 6AP, UK; ^3^Department of Microbiology, University of Lagos, Lagos 100223, Nigeria

## Abstract

Some rare strains of *Clostridium butyricum* carry the gene encoding the botulinal type E neurotoxin and must be considered as possible hazards in certain types of food. The limiting growth conditions for *C. butyricum* were determined in peptone yeast glucose starch (PYGS) broth incubated anaerobically at 30°C for up to 42 days. The minimum pH values permitting growth depended on the acidulant and strain. Organic acids were more effective at inhibiting growth than HCl as expected. The lowest pH values at which growth of toxigenic and nontoxigenic strains of *C. butyricum* was observed in broth acidified with HCl were 4.1 and 4.2, respectively. In organic acids, however, the minimum pH varied between 4.4 and 5.1 depending on acid type and concentration. The minimum water activity for growth of toxigenic strains of *C. butyricum* was 0.96. The minimum growth temperatures of the toxigenic strains of *C. butyricum* (ca 10-11°C) were somewhat higher than for non-toxigenic ones (8°C). It was concluded that control of toxigenic *C. butyricum* in the food industry needs to allow for the greater pH tolerance of this species compared with proteolytic *C. botulinum*.

## 1. Introduction

The main species of *Clostridium* associated with foodborne illness are *Clostridium botulinum* and *C. perfringens* and much effort has been directed towards defining the thermal treatments needed to inactivate their spores and the environmental conditions that will prevent their growth in food [1, 2]. Other Clostridia occur in certain types of food and; whilst they may cause serious spoilage problems, are not normally regarded as dangerous. Examples include *Clostridium tyrobutyricum* and other butyric Clostridia in cheese [3–7], *C. pasteurianum* and *C. butyricum* in canned fruit [8, 9], and the Clostridia that cause soft rot of vegetables [10]. Most strains of *C. butyricum* are harmless to humans and culture filtrates are not lethal to mice; in fact some strains have been investigated as probiotics for preventing diarrhoea [11–13], *Helicobacter pylori* infection [14], and inflammatory bowel disease [15]. However, some strains have acquired the type E botulinal neurotoxin gene (BoNT/E) and have caused both infant and classical foodborne botulism. The first recorded incident of *C. butyricum* type E botulism was a case of infant botulism in Italy [16]. Further cases of intestinal colonization by toxigenic *C. butyricum* strains were recorded in Italy but in older patients [17]. In 1994, an outbreak of botulism associated with *C. butyricum* occurred in the town of Guanyun, China, associated with consumption of a paste of fermented soybeans and melon. Two other outbreaks of botulism associated with *C. butyricum* were subsequently identified and were also linked to fermented soybeans [18, 19]. The long incubation period in some cases was suggestive of intestinal colonization. There has also been large botulism outbreak caused by *C. butyricum* type E in Gujarat, India, associated with Sevu a crisp made gram (chick-pea) flour [20].

Examination of soil samples taken from areas near the homes of patients of foodborne type E botulism in Guanyun and from five areas around lake Weishan yielded 20 type E toxin positive soil samples out of 60 tested samples [19]. *Clostridium butyricum* BoNT/E strains were isolated from four of the six sites surveyed. It was concluded that soil is a natural habitat of BoNT/E strains of *C. butyricum* as it is for *C. botulinum*. Genetic profiling of isolates from China and Italy suggested that BoNT/E producing *C. butyricum* is clonally distributed over a vast area [21] although attempts to isolate neurotoxigenic *C. butyricum* from soil around Rome were unsuccessful [22]. 

In a recent study, Ghoddusi and Sherburn [23] screened over a 100 food and environmental samples from the UK for type E producing *C. butyricum* and concluded that none of the isolates were in fact toxinogenic.

Implementation of HACCP in the food chain requires knowledge of hazard and the risk posed by foodborne microbes. In the case of *C. butyricum,* the hazard is the possible production of botulinum toxin in food or in the gut. The risk depends on the frequency with which toxigenic strains occurring in food materials and the likelihood that they are able to survive processing and grow and produce toxin. 

In our earlier study [23], the initial findings indicated differences with some of the previous studies in tolerance of toxigenic *C. butyricum* to growth limiting factors and therefore this study was conducted to examine how a different group of toxigenic and nontoxigenic strains of *C. butyricum* respond to limiting intrinsic and extrinsic conditions. Furthermore, most of such published studies date back decades ago, and some of the most recent ones were 20 years old and generating new data with new or different strains is necessary. 

As a result, the aim of this work was to define the conditions of pH, temperature, and solute concentration (sodium chloride and sucrose) that will prevent growth of some toxigenic in comparison to nontoxigenic strains of *Clostridium butyricum*.

## 2. Materials and Methods

### 2.1. Organisms

The strains of *Clostridium butyricum* used were as follows: ATCC 43755, ATCC 43181 (both type E neurotoxin producing strains as confirmed by the presence of BoNT/E gene in an earlier study by [23]), NCTC 7423, NCTC 6084, NCIMB 8082, and NCIMB 9575. Cultures were maintained at −70°C in Microbank vials (ProLab Diagnostics, Neston, Cheshire, UK).

### 2.2. Growth Media and Enumeration

Peptone-Yeast-Glucose-Starch Medium (PYGS medium) [24] containing resazurin (1.0 mg/L) as indicator was autoclaved in 500 mL volumes at 121°C for 15 min and transferred to an anaerobic cabinet (Don Whitley Scientific, Shipley, UK) containing a CO_2_/H_2_/N_2_ (5 : 10 : 85 v/v) atmosphere to cool overnight under reduced condition. Aliquots (9.5 mL) were transferred to Hungate tubes and sealed within the anaerobic cabinet. The tubes were then removed from the anaerobic cabinet and sterilised by autoclaving at 121°C for 15 minutes. PYGS Agar was sterilised at 121°C for 15 min and plates were poured under aseptic conditions, cooled, and stored at 4°C until required. Before use, plates were incubated for 24 h in an anaerobic cabinet. Samples were serially diluted in maximum recovery diluent (MRD) and plated on reinforced Clostridial agar (RCA, CM151) (Oxoid Ltd., Basingstoke, UK). Plates were incubated anaerobically at 30°C for 24 or 48 h.

### 2.3. pH and Growth Limit

The pH of the growth medium was adjusted with inorganic (HCl) and organic (acetic, lactic, and citric) acids. The pH of PYGS medium was adjusted with HCl (1 M), citric acid (1 M), acetic acid (5 M), or lactic acid (5 M) as required. An excess of this medium was prepared to allow pH to be measured in sample tubes of media immediately after autoclaving, immediately before use, and at the end of each 42-day experimental period. The pH meter was calibrated before the preparation of each batch of PYGS broth. The pH of the broth was adjusted in two ways. In the first method, acid was added to PYGS broth until the target value was achieved; in the second method the salt of the acid was added to a concentration of 50 mM, and the pH was then adjusted with HCl acid. It was not considered practical to compare acids at the same concentration of undissociated acid because the p*K*_a_ value for the dissociation of the last carboxyl group of citric acid is 3.14, which would mean adding unrealistically high concentrations of total acid to achieve reasonable levels of undissociated acid at relevant pH values (between 4 and 5). 

### 2.4. Solutes, Water Activity, and Growth Limit

Sodium chloride and sucrose solutions were used to study the limiting effects of water activity on growth of *C. butyricum* species.

### 2.5. NaCl

In initial experiments, sodium chloride was added to PYGS medium on a percentage weight to volume basis. This was later converted from w/v to w/w using a calibration curve. In later experiments, a weighed amount of salt was added to weighed medium to give w/w values. In all cases, the pH was adjusted to 7.0 prior to autoclaving and checked after autoclaving. All incubation was at 30°C. Corrections were made for the dilution effect of adding inoculum to the adjusted medium. Water activity was measured as described below.

### 2.6. Sucrose

Sucrose was added to PYGS medium on a percentage weight to volume basis, corrected for the dilution factor of adding 0.5 mL of PYGS medium without sucrose as described for NaCl (above). When preparing media containing sucrose, the sucrose and the solid constituents of PYGS medium were weighed out into a receptacle with volume gradations. Salt and antifoam solution (HyClone, Thermo Scientific, UK) were then added, followed by distilled water to the required volume. Media were weighed before and after the addition of media solutions and distilled water. This ensured that media concentration remained constant for the volume of medium prepared.

### 2.7. Water Activity Measurement

Water activity was measured using an Aqualab water activity meter (Labcell Ltd., Basingstoke, Hants, UK), calibrated with saturated salt solutions of known water activity. 

### 2.8. Temperature and Growth Limit

Experiments on the effect of temperature were conducted using a water bath incubated within an LMS cooled incubator. Temperatures within each water bath were monitored continuously with a Comark data logging thermometer using a type T thermocouple inserted into a Hungate tube containing 10 mL of water. The reading accuracy was approximately ±0.25°C. Tubes were incubated in the water bath, which acted as insulation against temperature fluctuations within the incubator.

### 2.9. Production of Spore Stocks

Spores of other species were produced by growth in PYGS broth or on PYGS plates. Growth from overnight cultures in PYGS broth was inoculated into PYGS broth or onto PYGA agar and incubated anaerobically at 30°C for 3-4 days (broth) or 7 days (agar). In some initial experiments, spores were produced using the two-phase Robertson's Cooked Meat Medium system as described by Peck et al. [25]. Sporulated growth on agar plates was suspended in sterile 0.85% (w/v) NaCl (saline). Spore suspensions or sporulated cultures were harvested by centrifugation at 15,000 ×g at 4°C for 15 min. Each pellet was washed 5 times with ice-cold sterile saline. The resultant pellet was suspended in 5–10 mL saline and stored at 4°C. For enumeration, spore suspensions were heat-shocked at 60°C for 10 minutes and plated onto reduced PYGS agar in triplicate. Inoculated plates were incubated at 30°C for 48 h under anaerobic conditions. The numbers of viable spores were then reported as colony-forming units (cfu) mL^−1^ of each spore suspension.

### 2.10. Defining the Growth Limits

An aliquot of each spore suspension was heat-shocked at 60°C for 10 min, cooled on ice and diluted in precooled deoxygenated PYGS broth. Growth of toxigenic strains *C. butyricum* ATCC 43755 and ATCC 43181 was studied individually. Spore suspensions of individual nontoxigenic strains were mixed to give final concentrations of 10^4^ to 2 × 10^6^ spores mL^−1^. Aliquots (500 *μ*L) of these suspensions inoculated into 9.5 mL of PYGS broth in Hungate tubes to give a final spore concentration of 10^2^–10^5^ spores mL^−1^. All experiments were conducted alongside negative controls inoculated with sterile PYGS broth and positive controls, where standard PYGS broth (pH 7.0 and 30°C incubation) was inoculated with spores. Each tube was examined daily for visible turbidity. Experiments were conducted over 42 days. Initially, three replicate tubes were inoculated at each pH or water activity and, if growth was observed within a few days, no further replicates were done at that value. If no growth was observed within 42 days, or if growth only occurred after more prolonged incubation, the experiment was repeated up to a further two to five times so that a total of 6–15 tubes were examined for each condition.

### 2.11. Detection of Growth

Visible turbidity occurs when cells reach a concentration of approximately 10^7^ cells mL^−1^ medium. Growth below this threshold would not be detectable by visual estimation. In order to determine the presence of growth below the visual threshold, 0.1 mL volume was taken from tubes at each concentration at time zero and after 1, 3, 5, 7, 14, 21, 28, 35, and 42 days of incubation. This aliquot was suspended in 9.9 mL Reinforced Clostridial Medium (RCM) and plated out onto Columbia blood agar. Plates were incubated under anaerobic conditions at 30°C for 48 h, and viable cell numbers were estimated by counting the colony forming units.

## 3. Results

### 3.1. Minimum pH for Growth

The limiting effect of pH on bacterial growth was measured using HCl, acetic, lactic, and citric acids. The lowest pH values at each growth observed are shown in Table 1. At pH values approaching the lower growth limit, not all replicate tubes showed growth and the results are based on the lowest pH value at which growth was observed in two to five separate trials in which triplicate tubes were inoculated at each pH value. In PYGS broth acidified with HCl the minimum pH for growth of toxigenic strains of *C. butyricum* was 4.2 whilst for the nontoxigenic strains the minimum pH was 4.1 (Table 1). In the presence of organic acids growth was inhibited at higher pH values. When compared at the same concentration of 50 mM, the effectiveness of the acids in preventing growth increased in the order lactic, acetic, and citric acid. In general, the two toxigenic strains of *C. butyricum *were inhibited at somewhat similar values to the nontoxigenic ones.

### 3.2. Minimum Water Activity for Growth

A range of solutes (NaCl and sucrose) concentrations were used to study the limiting effect of water activity on bacterial growth. The lowest water activity permitting growth of toxigenic strains of *C. butyricum* was 0.96 in either NaCl or sucrose. Growth of nontoxigenic strains of *C. butyricum* was possible at 0.96 in NaCl and 0.95 in sucrose (Table 2). The water activity values were rounded up or down to two decimal places. 

### 3.3. Minimum Temperature for Growth

The minimum temperature at which growth of *C. butyricum* was recorded was lower in the nontoxigenic strains (8°C) than in the toxigenic strains (10-11°C) (Table 2). 

## 4. Discussions 

The minimum pH values permitting growth of *C. butyricum* varied depending on the strain and the acidulant used to adjust the pH. Organic acids were more effective at inhibiting growth than HCl as generally found for other bacterial species. In general, the toxigenic strains of *C. butyricum* tended to be less tolerant of acidic conditions than the nontoxigenic ones; that is, they were inhibited at higher pH values as observed by others, though the differences were small and the trend did not hold true for all the conditions tested. 

The limiting pH values for growth of *C. butyricum* reported in previous studies are summarised in Table 3. The lowest pH at which growth of nontoxigenic strains of *C. butyricum* has been observed is pH 4.0 in bean sprout brine containing citric and lactic acids [26]. Morton et al. [27] observed growth of nontoxigenic *C. butyricum* strains in tomato juice at pH 4.2 but not at pH 4.0. These minimum pH values are quite close to the minimum pH in this work which was 4.1 in PYGS broth acidified with HCl, despite the differences in the acidulants used.

The lowest pH supporting growth of toxigenic strains of *C. butyricum* in this work was 4.2 in broth acidified with HCl, slightly higher than the pH limit for the nontoxigenic strains. The lowest pH values previously reported for growth of toxigenic strains were 4.8 [30] and 5.2 [27]. These differences in pH tolerance of toxigenic strains may perhaps be due to strain differences; the two toxigenic strains used in this work were ATCC 43181 and ATCC 43755, whilst Anniballi et al. [30] used a cocktail of strains from the Istituto Superiore di Sanita, Italy (CL20, CL21, CL86, CL109, CL145/1, CL146), and Morton et al. [27] used two strains (5262 and 5520) obtained from the Centers for Disease Control, Atlanta, Georgia, USA. The question of whether toxigenic strains of *C. butyricum* are generally less acid tolerant than nontoxigenic ones needs to be addressed by comparing all available toxigenic isolates with a range of nontoxigenic ones under the same conditions. The practical implication of these findings is that *C. butyricum* can grow at pH values below the cut-off point for growth of proteolytic stains of *C. botulinum* (pH 4.6) which is relevant when assessing the safety of processed foods where pH is the main or a significant controlling factor. Butyric acid producing anaerobes such as *C. butyricum*, *C. pasteurianum, *and *C. tyrobutyricum* have been associated with spoilage of a range of acid foods including canned pears, pineapples, tomatoes, fermented olives, and cheese. It is perhaps worth noting that *Clostridium pasteurianum* appears to be more acid tolerant than *C. butyricum* with growth being reported at pH 3.55 in 10% Brix canned pear juice and at pH 3.7 in 22% Brix apricot juice [8], so pH conditions that control butyric spoilage by *C. pasteurianum* will also prevent growth of toxigenic *C. butyricum* strains.

 The lowest water activities for growth of nontoxigenic strains of *C. butyricum* in this work were 0.95 in broth with sucrose and 0.96 in broth with NaCl. These values are lower than the values reported by Spiegelberg [31] (0.97–0.98 depending on solute) but similar to the minimum permitting growth of *C. pasteurianum* (0.95–0.96) (Table 4). 

The limit for toxigenic strains was 0.96 in both NaCl and sucrose as humectants. The limiting water activity for growth of proteolytic strains of *C. botulinum* is generally taken to be 0.94 [2] so water activities that prevent growth of proteolytic strains of *C. botulinum* will also ensure control of toxigenic *C. butyricum*. 

 The minimum growth temperatures of toxigenic *C. butyricum* strains (ca 10-11°C) are similar to those of proteolytic *C. botulinum* (10–12°C; [2]), although nontoxigenic strains were able to grow somewhat below this (8°C). The results are also in line with recent indication that the neurotoxigenic strains of *C. butyricum* require temperature greater than 12°C for growth [32]. 

*Clostridium butyricum* is a mesophilic anaerobic spore-forming organism and must therefore be considered as a possible hazard in foods that provide an anaerobic environment and are stored above 10–12°C. 

## 5. Conclusions

 The foods associated with reported outbreaks of *C. butyricum* type E botulism have been mainly of plant origin, that is, fermented soybeans in the Chinese outbreaks [20] and a crisp made from chick pea flour that had been improperly stored in the Indian outbreak [18, 19]. The food suspected of being involved in the Italian cases was canaderlo, a ball of bread, egg, cured ham parsley, and garlic that was heated in broth for a few minutes [30].

Where pH is a critical factor in food preservation, allowance must be made for the greater pH tolerance of *C. butyricum* compared with proteolytic *C. botulinum. *However, pH levels that prevent butyric spoilage in general should easily prevent growth of toxigenic *C. butyricum* strains in acid foods. Spores of butyric Clostridia may sometimes be found in nonacid foods such as milk products, and hence it is important to ensure that spores are eliminated from dairy-derived foods that will support their growth. 

## Figures and Tables

**Table 1:** Limiting pH values for growth of *Clostridium butyricum* in acidified PYGS broth.

Acidulant	Nontoxigenic *C. butyricum* cocktail	*C. butyricum* ATCC 43181	*C. butyricum* ATCC 43755
HCl	4.1 (1/9)^c^	4.2 (3/6)	4.2 (3/9)
Citric^a^	4.7 (6/12)	4.7 (5/12)	4.7 (3/12)
Lactic^a^	4.7 (1/6)	4.8 (3/6)	4.8 (6/6)
Acetic^a^	4.8 (2/9)	4.8 (2/9)	4.8 (2/9)
50 mM citric^b^	5.0 (4/6)	5.1 (6/6)	5.0 (3/6)
50 mM lactic^b^	4.6 (6/6)	4.4 (3/9)	4.4 (2/9)
50 mM acetic^b^	4.7 (3/12)	4.8 (3/12)	4.8 (6/9)

^a^pH adjusted by addition of concentrated acid to achieve target value.

^b^Final concentration (total acid) in the medium.

^c^Number of tubes positive within 42 days of incubation out of total inoculated at stated pH. Tubes at pH values 0.1 units lower were negative.

**Table 2:** Limiting water activities and temperatures for growth of *Clostridium butyricum* in acidified PYGS broth.

Limiting factor	Nontoxigenic *C. butyricum* cocktail	*C. butyricum* ATCC 43181	*C. butyricum* ATCC 43755
*a*_*w*_ (NaCl)	0.96 (3/15)^a^	0.96 (6/9)	0.96 (2/12)
*a*_*w*_ (Sucrose)	0.95 (6/12)	0.96 (8/9)	0.96 (4/6)
Temperature	8	10	11

^a^Number of tubes positive within 42 days of incubation out of total inoculated at stated water activity.

**Table 3:** Summary of some of the studies on growth-limiting pH values for *Clostridium butyricum* using organic and/or inorganic acids. Values are the lowest pH values where growth or no growth observed.

Strains	Growth medium	Acidulant	Growth	No growth	Reference
Non-toxigenic	Dextrose tryptone broth	Citric	4.4	4.2	Morton et al. [27]
	Broth		5.1	ND^a^	Bergère and Hermier^b^ [3]
	Bean sprout brine	Citric/lactic	4.0	3.7	De Jong [26]
	Pear juice^c^	Citric	—	>4.8	Jakobsen and Jensen [28]
	Tomato juice	HCl	4.2	4.0	Morton et al. [27]
	Liver infusion broth	Citrate-phosphate	4.5	4.3	Gilliland and Vaughn [29]

Toxigenic	TPYG broth	HCl	4.8	4.6	Anniballi et al. [30]
	Dextrose tryptone broth	Citric	5.2	5.0	Morton et al. [27]
	Tomato juice	HCl	ND	4.8	Morton et al. [27]

^a^Not determined.

^b^Inhibition of spore outgrowth.

^c^ *a*_*w*_ 0.990.

**Table 4:** Studies on limiting solute concentrations and water activity values permitting growth of *Clostridium butyricum*.

Strains	Temperature (°C)	Growth medium	Solute	Concentration (%w/v)	Water activity^a^	Reference
*C. butyricum *(non-toxigenic)	30	Broth pH 6.6	NaCl	<1.5–2.5	0.985	Bergère and Hermier^b^ [3]
	30	Tryptone glucose broth	Sucrose	28	0.977	Spiegelberg [31]
	30	Tryptone glucose broth	Glucose	15	0.98	Spiegelberg [31]
	30	Tryptone glucose broth	NaCl	2.3	0.985	Spiegelberg [31]
	30	Liver infusion broth	NaCl	5.0	0.97	Gilliland and Vaughn [29]

^a^Calculated from original concentration or osmotic pressure data.

^b^Inhibition of spore outgrowth.

## References

[1] Labbé RG, Lund BM, Baird-Parker A, Gould GW (1988). Clostridium perfringens. *The Microbiological Safety and Quality of Food*.

[2] Lund BM, Peck NW, Lund BM, Baird-Parker A, Gould GW (2000). Clostridium botulinum. *The Microbiological Safety and Quality of Food*.

[3] Bergère J-L, Hermier J (1970). Symposium on bacterial spores. XIV. Spore properties of clostridia occurring in cheese. *Journal of Applied Bacteriology*.

[4] Fowler GG (1979). The potential of nisin. *Food Manufacturing*.

[5] Senyk GF, Scheib JA, Brown JM, Ledford RA (1989). Evaluation of methods for determination of spore-formers responsible for the late gas-blowing defect in cheese. *Journal of Dairy Science*.

[6] Ryser ET (1999). Microorganisms of importance in raw milk. *Michigan Dairy Review*.

[7] Klijn N, Nieuwenhof FFJ, Hoolwerf JD, Van der Waals CB, Weerkamp AH (1995). Identification of Clostridium tyrobutyricum as the causative agent of late blowing in cheese by species-specific PCR amplification. *Applied and Environmental Microbiology*.

[8] Townsend CT (1939). Spore-forming anaerobes causing spoilage in acid canned foods. *Food Research*.

[9] Spiegelberg CH (1940). Some factors in the spoilage of an acid canned fruit. *Food Research*.

[10] Lund BM, Lund BM, Baird-Parker A, Gould GW (2000). Fresh and processed fruits. *The Microbiological Safety and Quality of Food*.

[11] Sato R, Tanaka M (1996). Multiplication of orally administered *Clostridium butyricum* in rats. *Microbial Ecology in Health and Disease*.

[12] Sato R, Tanaka M (1997). Intestinal distribution and intraluminal localization of orally administered *Clostridium butyricum* in rats. *Microbiology and Immunology*.

[13] Kamiya S, Taguchi H, Yamaguchi H, Osaki T, Takahashi M, Nakamura S (1997). Bacterioprophylaxis using *Clostridium butyricum* for lethal caedtis by *Clostridium difficile* in gnotobiotic mice. *Reviews in Medical Microbiology*.

[14] Takahashi M, Taguchi H, Yamaguchi H, Osaki T, Kamiya S (2000). Studies of the effect of *Clostridium butyricum* on Helicobacter pylori in several test models including gnotobiotic mice. *Journal of Medical Microbiology*.

[15] Kanauchi O, Mitsuyama K, Araki Y, Andoh A (2003). Modification of intestinal flora in the treatment of inflammatory bowel disease. *Current Pharmaceutical Design*.

[16] Aureli P, Fenicia L, Pasolini B (1986). Two cases of type E infant botulism caused by neurotoxigenic *Clostridium butyricum* in Italy. *Journal of Infectious Diseases*.

[17] Fenicia L, Franciosa G, Pourshaban M, Aureli P (1999). Intestinal toxemia botulism in two young people, caused by *Clostridium butyricum* type E. *Clinical Infectious Diseases*.

[18] Meng X, Karasawa T, Zou K (1997). Characterization of a neurotoxigenic *Clostridium butyricum* strain isolated from the food implicated in an outbreak of food-borne type *E. botulism*. *Journal of Clinical Microbiology*.

[19] Meng X, Yamakawa K, Zou K (1999). Isolation and characterisation of neurotoxigenic *Clostridium butyricum* from soil in China. *Journal of Medical Microbiology*.

[20] Chaudhry R, Dhawan B, Kumar D (1998). Outbreak of suspected *Clostridium butyricum* botulism in India. *Emerging Infectious Diseases*.

[21] Wang X, Maegawa T, Karasawa T (2000). Genetic analysis of type *E. botulinum* toxin-producing *Clostridium butyricum* strains. *Applied and Environmental Microbiology*.

[22] Creti R, Fenicia L, Aureli P (1990). Occurrence of Clostridium botulinum in the soil of the vicinity of Rome. *Current Microbiology*.

[23] Ghoddusi HB, Sherburn R (2010). Preliminary study on the isolation of *Clostridium butyricum* strains from natural sources in the UK and screening the isolates for presence of the type E botulinal toxin gene. *International Journal of Food Microbiology*.

[24] Lund BM, Wyatt GM, Graham AF (1985). The combined effect of low temperature and low pH on survival of, and growth and toxin formation from, spores of Clostridium botulinum. *Food Microbiology*.

[25] Peck MW, Fairbairn DA, Lund BM (1992). The effect of recovery medium on the estimated heat-inactivation of spores of non-proteolytic Clostridium botulinum. *Letters in Applied Microbiology*.

[26] de Jong J (1989). Spoilage of an acid food product by Clostridium perfringens, *C. barati* and *C. butyricum*. *International Journal of Food Microbiology*.

[27] Morton RD, Scott VN, Bernard DT, Wiley RC (1990). Effect of heat and pH on toxigenic *C. butyricum*. *Journal of Food Science*.

[28] Jakobsen M, Jensen HC (1975). Combined effect of water activity and pH on the growth of butyric anaerobes in canned pears. *Lebensmittel-Wissenschaft Technologie*.

[29] Gilliland JR, Vaughn RH (1943). Characteristics of butyric acid bacteria from olives. *Journal of Bacteriology*.

[30] Anniballi F, Fenicia L, Franciosa G, Aureli P (2002). Influence of pH and temperature on the growth of and toxin production by neurotoxigenic strains of *Clostridium butyricum* type E. *Journal of Food Protection*.

[31] Spiegelberg CH (1944). Sugar and salt tolerance of Clostridium pasteurianum and some related anaerobes. *Journal of Bacteriology*.

[32] Glass K, Marshal K, Morris JG, Potter ME (2013). Clostridium botulinum. *Foodborne Infections and Intoxications*.

